# Green-Synthesized titanium dioxide Nanoparticle–Modified glass ionomer cement: in vitro and in Silico assessment of Mechanical, Physical, and safety properties performance

**DOI:** 10.1038/s41598-026-37048-2

**Published:** 2026-02-10

**Authors:** Dina Abozaid, Abdullah Ayad, Yomna Ibrahim, Amr Azab, Mohamed Abd El-Aal, Samy El-Safty

**Affiliations:** 1 Dental Biomaterials Department, Faculty of Dentistry, Tanta University, El-Geish Street, Tanta, 31511 Egypt; 2 College of Dentistry, University of Basrah, Basra, Iraq; 3 Molecular Genetics and Proteomics Department, Bayan National Lab for Advanced Medical Diagnostics, Basra, Iraq; 4 Dental Biomaterials Department, Faculty of Dentistry, Alexandria University, Alexandria, Egypt; 5 Prosthodontics Department, Faculty of Dentistry, Tanta University, Tanta, Egypt; 6 Chemistry Department, Faculty of Science, Assiut University, Assiut, 71516 Egypt

**Keywords:** Glass ionomer cement, Phytotherapeutics, Flexural strength, Vickers hardness, Titanium dioxide nanoparticles, *Citrus aurantium L.* seed extract, Chemistry, Materials science, Nanoscience and technology

## Abstract

Recurrent caries continues to pose a major problem in restorative dentistry. Although numerous antimicrobial agents have been added to restorative materials, their effectiveness and their influence on the materials’ mechanical properties are still uncertain. This study investigated the physicomechanical performance of glass ionomer cement (GIC) modified with green-synthesized titanium dioxide nanoparticles prepared using Citrus aurantium seed extract (CA-TiO₂NPs). The novelty of this work lies in integrating a plant-mediated synthesis approach with systematic mechanical evaluation and preliminary in silico safety screening within a single proof-of-concept framework. CA-TiO₂NPs were synthesized via phytoreduction using C. aurantium seed extract and added to the powder phase of a conventional GIC at 5% and 10% w/w. Flexural strength was evaluated according to ISO 9917-2:2017, water sorption and solubility according to ISO 4049:2009, and Vickers microhardness according to ASTM E384. The major bioactive constituents identified by Gas Chromatography-Mass Spectrometry (GC–MS) were profiled. In addition, computational toxicological and ADME predictions were performed to provide early-stage safety indicators. While nanoparticle incorporation influenced selected mechanical parameters, flexural strength differences were not statistically significant. The 10% CA-TiO₂ NPs group exhibited the highest flexural modulus and microhardness (P < 0.0001) and lowest water sorption and solubility. Additionally. In silico pharmacokinetic and toxicological analyses suggested a favorable preliminary safety profile of the phytochemical constituents, supporting proof-of-concept evaluation rather than clinical validation. The combination of green synthesis and mechanical testing, together with screening-level toxic informatics of major extract-associated metabolites, supports a proof-of-concept for improving CA TiO₂ NPs –modified GIC properties while prioritizing safety-relevant endpoints for subsequent leachate and cytotoxicity testing. Clinical Significance: Computational integration strengthens the translational validity of green nanomaterial development by providing preliminary molecular-level safety indicators for phytochemical constituents potentially associated with the modified material.

**Supplementary Information:**

The online version contains supplementary material available at 10.1038/s41598-026-37048-2.

## Introduction

Glass ionomer cements (GICs) have been extensively used in dentistry for over half a century, serving as liners, bases, luting agents, sealers, and restorative materials^1^. They remain a material of choice due to their ability to bond chemically to tooth structures, inherent antimicrobial activity through fluoride release, thermal compatibility, and favorable biocompatibility^2^. Despite these advantages, GICs present notable limitations, including sensitivity to moisture, intrinsic opacity, wear susceptibility, and suboptimal mechanical strength^2^. While their self-curing properties and potential for caries inhibition are well established, their ability to prevent secondary caries entirely remains questionable^3,4^. Failed restorations can result in edentulism, compromising mastication, speech, aesthetics, and overall oral health, with significant effects on quality of life^5–10^. To address GIC limitations, various antimicrobial agents, including chlorhexidine (CHX), have been incorporated, though concerns about their adverse effects on mechanical properties persist^11^. The performance of restorative materials is influenced by parameters such as hardness, water sorption, solubility, and flexural strength^12–14^ with water sorption and solubility being particularly critical for cement stability.

In parallel, global interest in herbal and naturally derived antimicrobial agents has surged^4,15^ with green nanotechnology, convergence of phytochemistry and nanoscience offering a sustainable pathway to develop bioactive dental materials^16^. Plant extracts rich in secondary metabolites, such as flavonoids, phenols, alkaloids, terpenoids, and limonoids, act as natural reducing and stabilizing agents in nanoparticle synthesis^17^. Citrus species, belonging to the *Rutaceae* family, are well known for their nutritional and medicinal value, being abundant in vitamins, minerals, and phytochemicals with strong antioxidant and antimicrobial properties^18,19^. *Citrus aurantium* (bitter orange) seed extract has reported richness in phenolic compounds, fatty acids, and reducing agents capable of facilitating nanoparticle formation while simultaneously acting as stabilizing agents^20–22^. Unlike peel-based extracts, seed extracts offer a distinct phytochemical profile that may influence nanoparticle nucleation and surface chemistry.

Recent studies have demonstrated the feasibility of green-synthesized metal and metal oxide nanoparticles using plant extracts, highlighting their structural tunability and potential functional applications. Notably, recent reports have explored environmentally benign synthesis routes and advanced characterization strategies, underscoring the growing interest in sustainable nanomaterial development^23–28^.

Specific phytochemicals such as phenolic acids, flavonoids, and fatty acids are known to participate in nanoparticle formation by acting as electron donors and stabilizing ligands, facilitating nucleation and growth control during green synthesis. Previous reports demonstrate that such compounds can adsorb onto metal oxide surfaces, modulating particle size, crystallinity, and dispersion, which in turn influence material properties when integrated into polymeric matrices^25,29,30^.

Leveraging these bioactives in nanoparticle synthesis represents a promising strategy for advancing dental biomaterials. Previous studies incorporating plant-mediated nanoparticles into GICs, such as silver nanoparticles synthesized using *Zingiber officinale*^31^, hydroxyapatite nanoparticles from *Moringa oleifera*^32^, and silver nanoparticles from *Salsola imbricata*^33^, have demonstrated improved mechanical strength, reduced porosity, and enhanced antibacterial effects.

In silico methods are increasingly used in materials research to screen potential toxicological alerts of candidate organic constituents that could be released under degradation or wear scenarios, using toxicoinformatics models originally developed for small-molecule pharmacokinetics. Tools such as SwissADME, Deep-PK, and ProTox-3.0 permit rapid evaluation of physicochemical, absorption, metabolism, and toxicity descriptors for plant metabolites that may remain embedded or bound within modified biomaterials^34,35^. Integrating such models strengthens experimental validation by linking molecular properties to safety expectations at the preclinical stage^35,36^.

Therefore, this study was conducted to introduce dual experimental and in silico design. Titanium dioxide nanoparticles were synthesized using aqueous *Citrus aurantium* seed extract and incorporated into commercial GIC at 5% and 10% w/w concentrations. The mechanical (flexural and hardness), hydric (sorption and solubility), and computational toxicological properties of the system were systematically assessed. Besides mechanical enhancements, the evaluation of safety properties through in silico modeling of the phytochemical constituents is useful as an early-stage prioritization step to identify any toxicity alerts among major metabolites and to guide follow-up testing (leachate characterization and standardized cytotoxicity assays), thereby advancing the clinical translatability of green-synthesized nanoparticles in dental biomaterials.

Although plant-mediated TiO₂ nanoparticle modifications of dental materials have been reported, limited studies integrate systematic in vitro mechanical evaluation with in silico toxicological screening to contextualize material safety. This study addresses this gap by combining experimental performance testing with computational safety prediction as a proof-of-concept framework. The null hypothesis stated that incorporation of Citrus aurantium-mediated TiO₂ nanoparticles into glass ionomer cement would not result in statistically significant differences in flexural strength, elastic modulus, surface microhardness, water sorption, or solubility compared with the unmodified material.

## Methods

### Preparation of *Citrus aurantium* (CA) seeds extract

*Citrus aurantium* was cultivated and harvested in the spring of 2024 in Nubaria, Egypt. To maintain the quality of the CA seeds and preserve their active constituents, the seeds were air-dried for two weeks, followed by additional drying in a vacuum oven at 40 °C with calcium fluoride as a desiccant to achieve complete dehydration. After drying, the seeds were ground into a fine powder using an electric blender^37^. For the extraction process, 20 g of powdered seeds were boiled in 200 mL of distilled water for 2 h^38^^39^, to promote cell wall disruption and enhance the release of water-soluble phytochemicals. Distilled water was selected as a green, non-toxic solvent compatible with subsequent synthesis steps. After cooling to room temperature, the mixture was filtered repeatedly to remove residual solids, and the obtained extract was stored under refrigeration for further use^37^.

### Characterization of *Citrus aurantium* L. Seed extract

#### Phytochemical screening of *Citrus aurantium* L. Seed extract

The phytochemical analysis of *Citrus aurantium* L. seed extract identified key bioactive compounds, including coumarins, steroids, flavonoids, tannins, alkaloids, and phenols using standardized qualitative methods^40–44^.

- Coumarins were confirmed by a yellow color change with NaOH.
- Steroids were identified by a red hue in chloroform upon the addition of H₂SO₄.
- Flavonoids were detected by a buff precipitate with lead acetate.
- Tannins produced a greenish-black color upon FeCl₃ addition.
- Alkaloids were verified by a cream or brown-red precipitate with Mayer’s reagent.
- Phenols were detected by a greenish-blue precipitate with ferric chloride and potassium ferricyanide.

This comprehensive analysis established the phytochemical profile of the *Citrus aurantium* L. seed extract.

#### Gas Chromatography-Mass spectrometry (GC-MS) analysis of CA seed extract

Gas chromatography–mass spectrometry (GC/MS) analysis was performed using an Agilent Technologies 7890 A gas chromatograph system (Agilent, HP-6890, USA) equipped with an HP-5ms fused silica column (30.0 m × 0.25 mm × 0.25 μm). The oven temperature program was started at 50 °C (held for 1 min), increased by 10 °C per minute to 125 °C, and subsequently reached 250 °C (held for 5 min) at a rate of 5 °C per minute. The injector and detector temperatures were set at 200 °C and 250 °C, respectively.

Approximately 1 mL of *Citrus aurantium* L. was diluted in diethyl ether and injected, with helium as the carrier gas at a flow rate of 1.0 mL per minute. The GC/MS system operated in Electron Ionization (EI) mode at 70 eV ionization potential, scanning a mass range of 40–440 m/z in positive mode. The interface temperature was set at 250 °C.

Active components were identified by comparing mass spectra with the National Institute of Standards and Technology (NIST) and WILEY libraries. Percentage compositions were calculated based on peak area relative to the total area. Compounds were preliminarily identified by comparing their mass spectra and retention times with authentic standards and literature-reported data. Fragmentation patterns were also matched with previously reported information to confirm compound identification^45,46^.

### Green synthesis of TiO₂ nanoparticles using CA extract (CA-TiO₂ NPs)

Approximately 100 mL of titanium isopropoxide solution was added dropwise to the Citrus aurantium seed extract under vigorous stirring at 80 °C, enabling controlled hydrolysis of the precursor mediated by bioactive phytochemicals in the extract. The effective precursor-to-extract ratio was maintained constant in all synthesis batches to ensure reproducibility. The reaction pH was not externally adjusted and was instead governed by the intrinsic acidity of the extract, consistent with green synthesis approaches that avoid chemical additives. The mixture was aged for 2 h to facilitate nanoparticle nucleation and growth. The resulting yellowish-white precipitate was separated by centrifugation at 5,000 rpm for 10 min, washed repeatedly with distilled water to remove unreacted species, and re-centrifuged. The collected precipitate was dried overnight at 80 °C and subsequently calcined at 500 °C to obtain TiO₂ nanoparticles^47^.

### Characterization of (CA-TiO₂ NPs)

The structure and crystallinity of TiO₂ NPs were analyzed using X-ray diffraction (XRD) on a Philips diffractometer (description) (PW 2103, Cu Kα, λ = 1.5418 Å) over a 4°−80° range. The average crystallite size of the TiO₂ NPs, calculated using the Scherrer Eqs. 4^8^^49^, Fourier-transform infrared spectroscopy (FTIR) was employed to identify the chemical structures and functional groups using a Nicolet spectrophotometer (Model 6700) in the 4000–400 cm⁻¹ range^50^. Thermal stability of CA-TiO₂ NPs was evaluated using a thermogravimetric analyzer (TGA, PerkinElmer, TGA4000, USA) with a temperature range from ambient temperature to 800 °C^51^. The morphology of the TiO₂ NPs was characterized using a transmission electron microscope (TEM, JEOL, 2100 F, Japan).

### Preparation of glass ionomer cement modified with CA-mediated TiO₂ nanoparticles

CA-mediated TiO₂ nanoparticles were incorporated into the powder phase of conventional glass ionomer cement at concentrations of 5 wt% and 10 wt%, replacing an equivalent mass of GIC powder. The liquid-to-powder ratio was maintained according to the manufacturer’s instructions (Fuji IX GP, GC Corporation, Tokyo, Japan)^52^. The grouping was as follows:

1. Control Group: GIC without CA-mediated TiO₂ nanoparticles.
2. 5% CA-TiO₂ NPs Group: Modified GIC containing 5% CA-mediated TiO₂ nanoparticles.
3. 10% CA-TiO₂ NPs Group: Modified GIC containing 10% CA-mediated TiO₂ nanoparticles.

### Flexural strength measurement

For flexural strength testing, thirty bar-shaped specimens (*n* = 10 per group) measuring 2 × 2 × 25 mm were fabricated. After packing the GIC mixtures into a Teflon mold, a glass microscope slide was positioned on top and gentle hand pressure was applied^17,37^. Once the specimens had set, they were removed from the mold, and any excess material was trimmed using 800-grit silicon carbide paper. All samples were then stored in distilled water at 37 °C for 24 h^17,37^. Flexural strength was determined using a three-point bending setup on an INSTRON 3345 universal testing machine (Norwood, MA, USA), with a 20 mm support span and a crosshead speed of 1 mm/min. The flexural strength (FS) and flexural modulus (FM) were calculated using the following formulas:

FS = 3FL/(2bd²).

FM = FL³/4bd³.

where (F) is the force in Newton, (L) is the span length in mm, (b) is the specimen width in mm, and (d) is specimen thickness in mm.

### Vickers Micro-Hardness measurement

For microhardness evaluation, thirty disc-shaped specimens (*n* = 10 per group) measuring 8 × 2 mm were prepared following the manufacturer’s instructions. After setting, the specimens were demolded and polished with 800-grit silicon carbide paper to standardize the surfaces and eliminate excess material. The polished samples were then stored in distilled water at 37 °C for 24 h to simulate pre-testing conditions^17,37^.

Surface microhardness was measured using a Digital Display Vickers Microhardness Tester (Model HVS-50, Laizhou Huayin Testing Instrument Co., Ltd., China) equipped with a Vickers diamond indenter and a 20× objective lens. A load of 100 g was applied for 15 s on each specimen. To ensure reliability, three indentations were made per specimen, evenly distributed and spaced at least 0.5 mm apart from one another^52–54^.

### Water sorption and solubility

A total of 30 disc-shaped specimens, each 15 mm in diameter and 1 mm thick, were fabricated using a Teflon mold (*n* = 10 per group). For each group, the material was mixed following the manufacturer’s instructions and placed into the mold on a glass slab. A polyester matrix strip was positioned over the surface and lightly pressed with a glass slide until setting was complete. Any specimens that were malformed or contained voids were excluded^55^.

After one hour, specimens were placed in a desiccator containing silica gel (Merck KGaA, Darmstadt, Germany) for 2 h, followed by incubation in an oven at 37 °C for 22 h to obtain a constant mass, with an acceptable weight variation of no more than ± 0.0005 g. Specimens were then weighed using a precision analytical balance (JP105DUG, Mettler-Toledo GmbH, Giessen, Germany) to determine their initial mass (m1).

Each specimen was immersed in a labelled container filled with 25 mL of deionized water and stored in an incubator at 37 °C for 7 days. After immersion, specimens were removed, gently dried with filter paper, and re-weighed to obtain the mass after immersion (m2). The samples were then dehydrated in an incubator at 37 ± 1 °C for 24 h and weighed again to record the final mass after dehydration (m3).

Solubility and sorption were calculated using the following Eq^55^.:

Solubility = (M1 - M3)/V.

Sorption = (M2 - M1)/V.

Where (V) is the specimen volume in ml.

### In Silico analysis

Ten major GC–MS–identified phytochemicals from the *Citrus aurantium* seed extract reported in the manuscript were evaluated in silico. Canonical SMILES for each compound were retrieved from PubChem. SwissADME was used first to calculate key physicochemical and ADME descriptors, including consensus logP, TPSA, ESOL solubility class, GI absorption, BBB permeation, P-gp substrate status, CYP1A2/2C19/2C9/2D6/3A4 inhibition flags, skin permeation (logKp), standard drug-likeness filters (Lipinski, Veber, Egan, Ghose, Muegge), bioavailability score, PAINS/Brenk alerts, lead-likeness, and synthetic accessibility^34^. Next, Deep-PK was applied to the same SMILES to estimate absorption (Caco-2, oral bioavailability 50%), distribution (BBB penetration, plasma-protein binding), excretion (total clearance, half-life class), and selected safety panels^35^. Finally, ProTox-3.0 was used to predict oral LD₅₀/class and binary calls for major organ toxicities, carcinogenicity, mutagenicity, cardiotoxicity, immunotoxicity, neurotoxicity, and selected nuclear-receptor/stress-response endpoints^36^. Outputs were recorded exactly as reported by each server and compiled into summary tables for interpretation alongside the material performance data.

### Statistical analysis

Normality was verified using the Shapiro–Wilk test. Statistical analysis was performed using OriginPro 2025 (OriginLab Corporation, USA) and significance level was set at *P* ≤ 0.05. One-way ANOVA was employed to detect the difference between study groups followed by Tukey post hoc test with Bonferroni.

## Results

### Phytochemical screening

The biochemical analysis of *Citrus aurantium* L. aqueous seed extract revealed the presence of four bioactive components: coumarins, alkaloids, phenols, and flavonoid derivatives, while results were negative for steroids, and tannins.

### GC/MS analysis of CA seeds extract

**Fig. 1:**
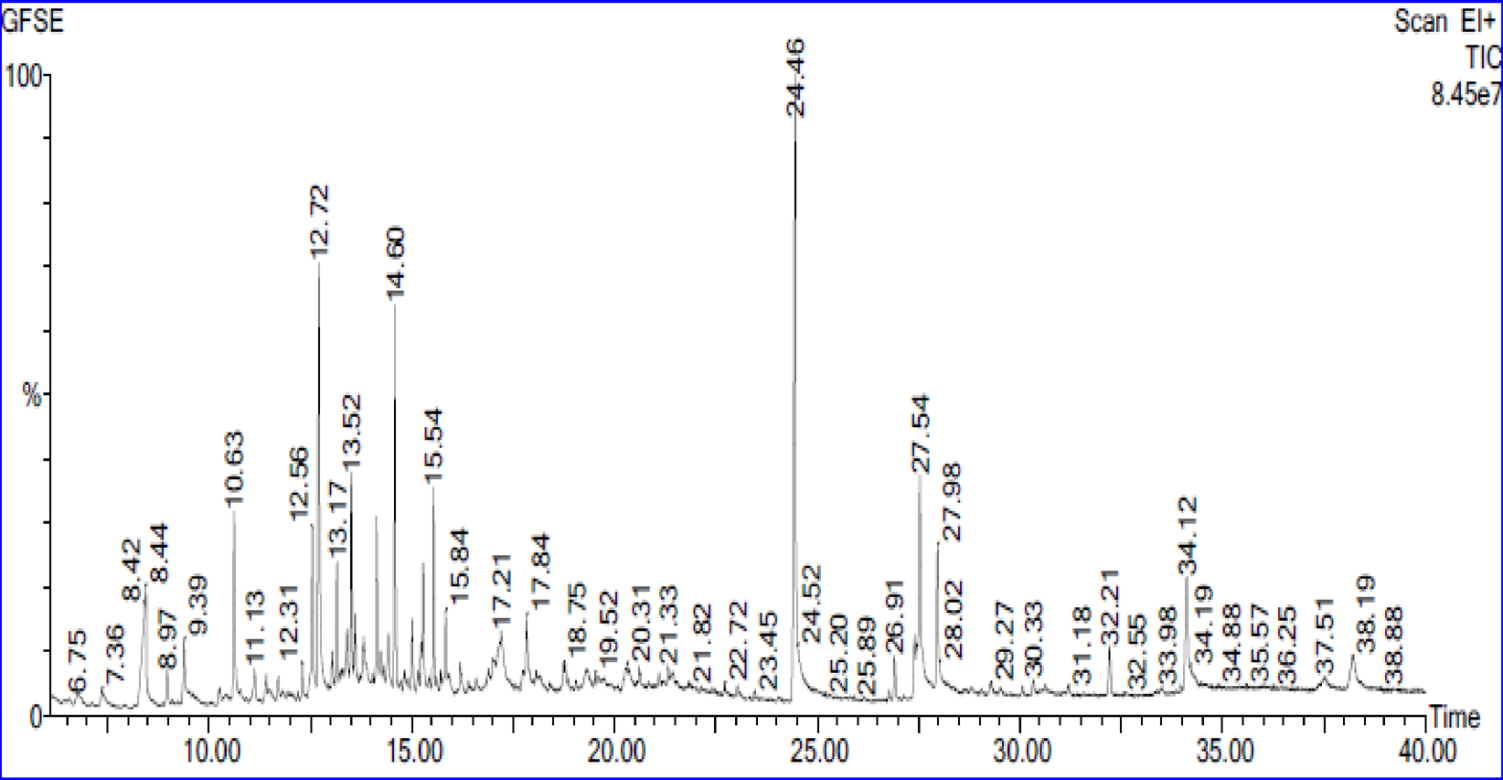
GC-MS spectral chromatogram of *Citrus aurantium L. (bitter orange)* seed extract.

The phytochemical components of CA aqueous seed extract were identified using GC-MS analysis. The GC-MS spectral chromatogram of the CA extract is presented in Fig. 1. The GC-MS data revealed the presence of 50 components, most of which are fatty acids. The ten major components identified in the acetone seed extract of *Citrus aurantium* are in Table 1.

**Table 1:** Main phytochemical components of *C. aurantium L.*(bitter orange) seed extract identified by GC-MS^37^.

Compound Name	Molecular Formula	RT (min)	Area %
n-Hexadecanoic acid	C_16_H_32_O_2_	24.461	11.247
Cis-Vaccenic acid	C_18_H_34_O_2_	27.537	4.161
2-Furancarboxaldehyde, 5-(hydroxymethyl)-	C6H6O3	12.716	4.789
Thymine	C_5_H_6_N_2_O_2_	8.439	3.964
2,4-Decadienal (E, E)	C_10_H_16_O	14.597	3.180
Hexadecanoic acid, 2-hydroxy-1-(hydroxymethyl)ethyl ester methyl-	C_19_H_38_O_4_	34.119	3.491
Octadecanoic acid	C_18_H_36_O_2_	27.977	2.359
2-Nitrohept-2-en-1-ol	C_7_H_13_NO_3_	17.208	2.737
4 H-Pyran-4-one, 2,3-dihydro-3,5-dihydroxy-6-methyl-	C_6_H_8_O_4_	10.625	2.431
Benzofuran, 2,3-dihydro-	C_8_H_8_O	12.556	2.083

### X-Ray diffraction (XRD)

**Fig. 2:**
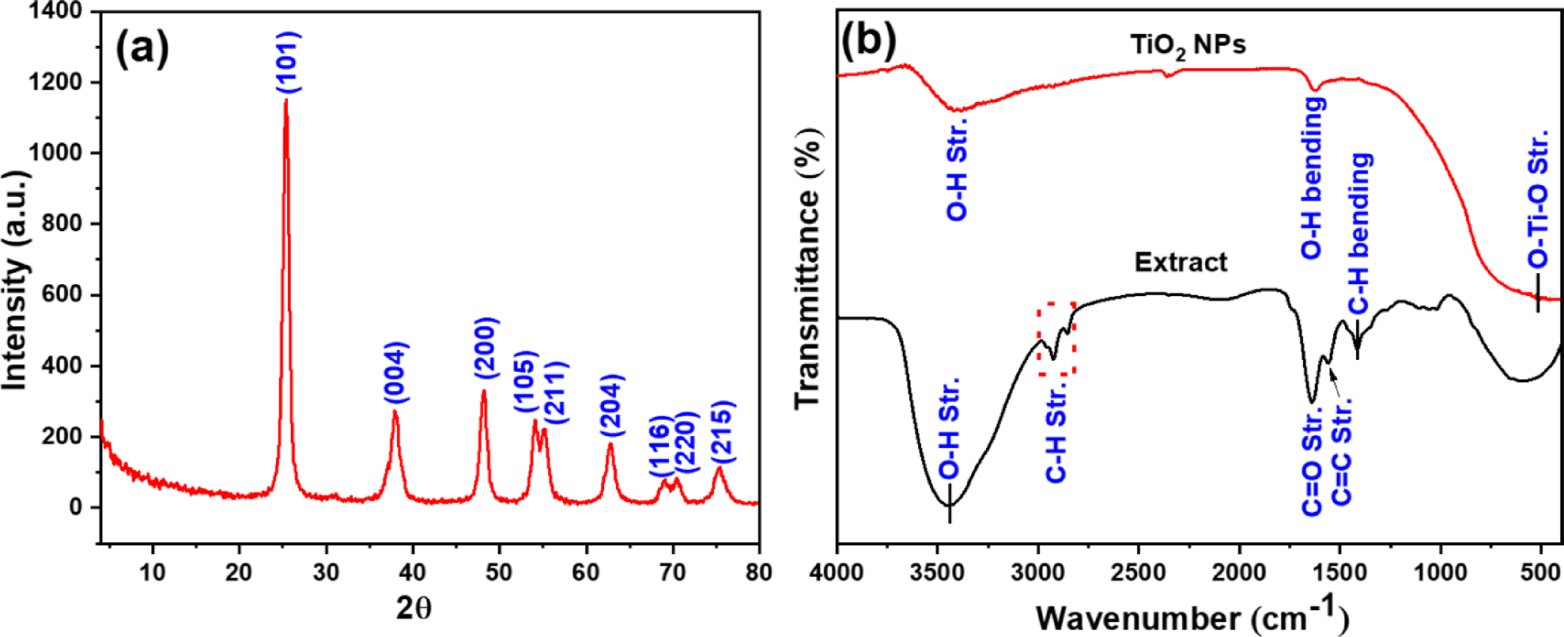
(**a**) XRD pattern of TiO_2_ NPs, and (**b**) FTIR spectra of plant extract and TiO_2_ NPs.

The XRD diffraction pattern of TiO₂ NPs is displayed in Fig. 2a. The prepared sample exhibited diffraction peaks at 2θ values of 25.3°, 37.9°, 48.2°, 54.1°, 55.2°, 62.7°, 69.1°, 70.4°, and 75.4°, corresponding to the (101), (004), (200), (105), (211), (204), (116), (220), and (215) planes of tetragonal anatase TiO₂ NPs (JCPDS No. 01–084–1286), respectively. The average crystallite size of the TiO₂ NPs was determined to be 15.5 nm.

### **Fourier Transform Infrared (FTIR) Spectroscopy**

The FTIR spectra of the CA extract and TiO₂ NPs are shown in Fig. 2bThe FTIR spectrum of the CA extract displayed absorption bands at approximately 3400 cm⁻¹ (O-H stretching), 1639 cm⁻¹ (C = O stretching), and 1554 cm⁻¹ (C = C stretching), corresponding to hydroxyl, carbonyl, and alkenyl groups, respectively. In addition, the band that located at 1418 cm⁻¹ is attributed to the C-H bending of aromatic phenolic compounds. The FTIR spectrum of the TiO_2_–NPs sample exhibits characteristic bands at 3384 cm⁻¹ corresponding to O–H stretching vibrations, 1624 cm⁻¹ assigned to O–H bending of adsorbed water, and 530 cm⁻¹ attributed to O–Ti–O stretching vibrations.

### Transmission electron microscopy (TEM)

**Fig. 3:**
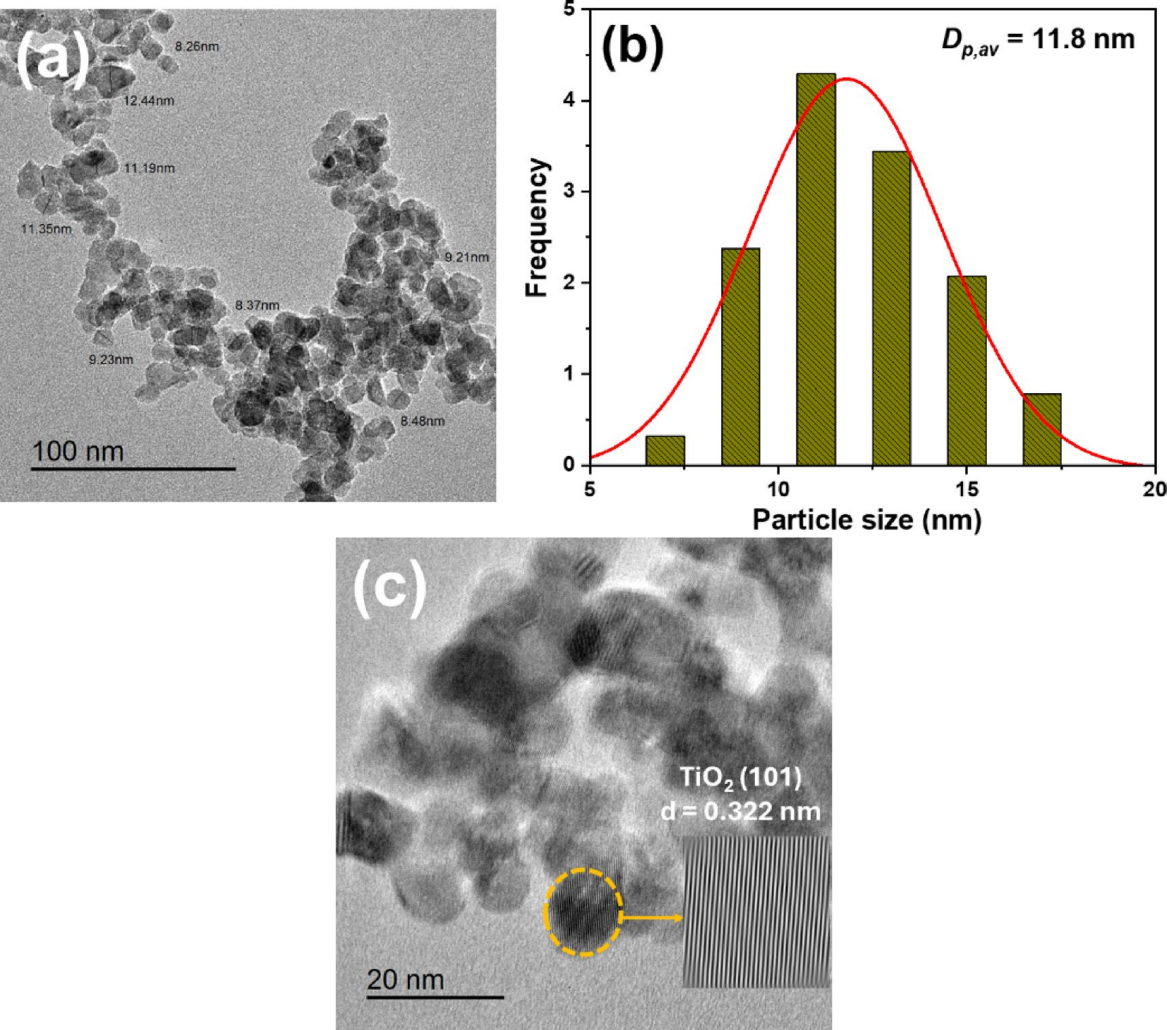
(**a**) TEM image, (**b**) particle size distribution, and (**c**) HR-TEM image of CA-TiO_2_ NPs.

The morphology and size distribution of the CA-TiO₂NPs were examined using HR-TEM and the results are shown in Fig. 3. The TEM image revealed that the nanoparticles were quasi-spherical in shape (Fig. 3a). The particle size distribution analysis (Fig. 3b) showed that the CA-TiO₂ NPs with an average size of 11.8 nm. The HR-TEM image of TiO_2_ NPs (Fig. 3c) showed a lattice spacing of 0.322 nm corresponding to the d-spacing of TiO_2_ (101).

### Thermogravimetric analysis (TGA)

**Fig. 4:**
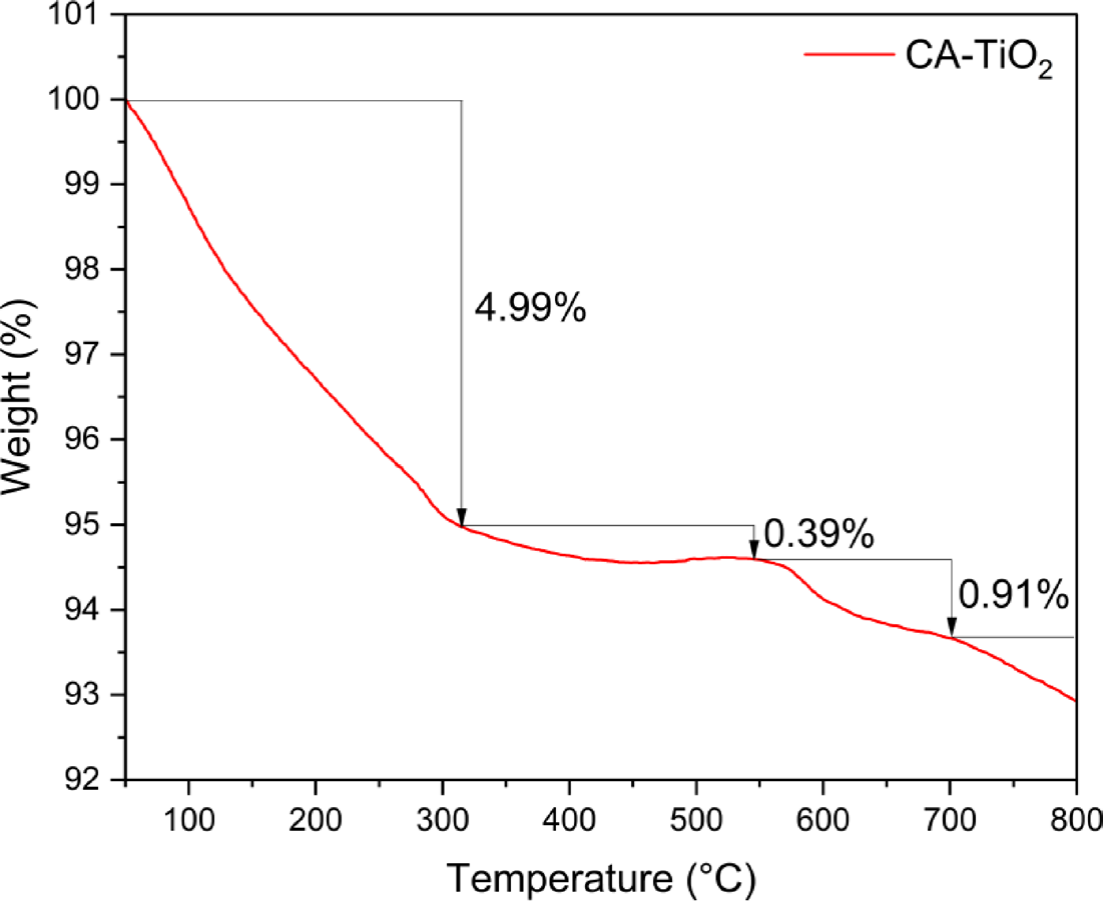
Thermogravimetric analysis of CA-TiO_2_ NPs.

Thermogravimetric analysis of CA-TiO₂NPs showed a three-step mass-loss profile with a cumulative weight reduction of ≈ 6.29% between 50 °C and 800 °C (Fig. 4).

### Flexural strength results

**Fig. 5:**
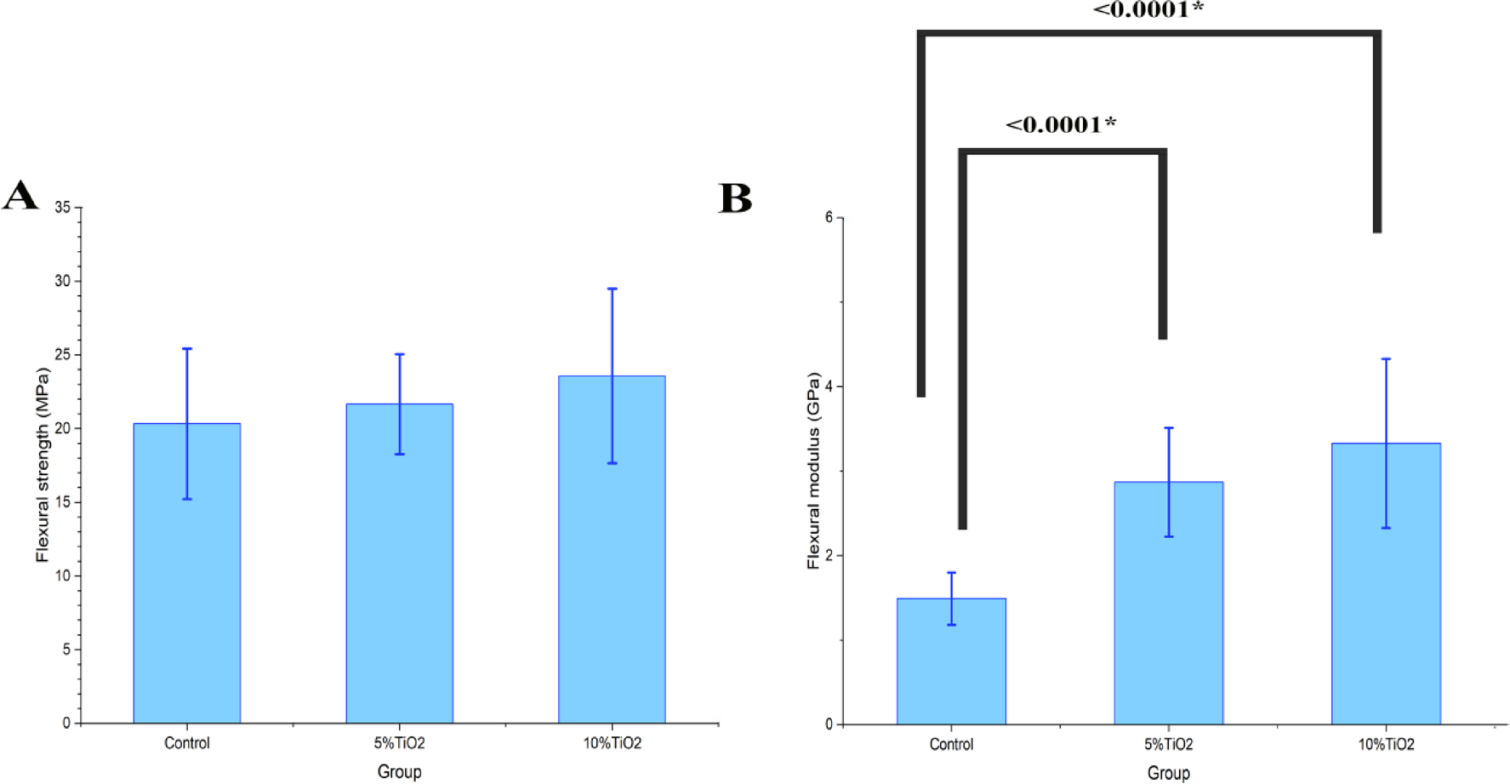
Comparisons of mean and SD of **(A)** flexural strength (MPa) and **(B)** flexural modulus (GPa) between study groups.

The 10% CA-TiO₂ NPs group showed the highest flexural strength (23.6 ± 5.6 MPa) (Fig. 5A)and flexural modulus (3.3 ± 1.0 GPa) compared to the other groups (Table 2). Flexural strength values showed no statistically significant differences among groups (*P* = 0.35) (Fig. 5A), whereas flexural modulus exhibited statistically significant differences (*P* < 0.0001) (Table 3). Regarding the flexural modulus, the control and the 5% CA-TiO₂ NPs as well as the control with 10% CA-TiO₂ NPs showed a statistically significant difference at *P* < 0.0001 (Fig. 5B).

**Table 2:** Descriptive values of mean, standard deviation, standard error, and confidence intervals of flexural strength, flexural modulus, Vickers microhardness, sorption, and solubility of study groups.

Property	Parameter	Control	5% CA-TiO₂	10% CA-TiO₂
**Flexural strength**	**Mean (MPa)**	20.3	21.7	23.6
	**SD**	5.1	3.4	5.6
	**SE**	1.6	1.1	1.8
	**CI 95%**	17.3–23.3	19.7–23.7	20.1–27.1
**Flexural modulus**	**Mean (GPa)**	1.5	2.9	3.3
	**SD**	0.3	0.6	1.0
	**SE**	0.1	0.2	0.3
	**CI 95%**	1.3–1.7	2.5–3.2	2.7–3.9
**Vickers microhardness**	**Mean (VHN)**	78.5	82.1	100.4
	**SD**	7.5	6.8	5.9
	**SE**	2.4	2.1	2.0
	**CI 95%**	74.1–82.9	78.1–86.0	96.7–104.0
**Sorption**	**Mean (µg/ml)**	3.2	2.2	2.0
	**SD**	0.7	0.3	0.1
	**SE**	0.2	0.1	0.1
	**CI 95%**	2.8–3.7	2.1–2.4	1.9–2.1
**Solubility**	**Mean (µg/ml)**	−5.5	0.5	0.2
	**SD**	0.6	0.4	0.1
	**SE**	0.2	0.1	0.1
	**CI 95%**	−5.9 – (−5.1)	0.3–0.8	0.1–0.2

SD, standard deviation; SE, standard error; CI, confidence interval; MPa, Mega pascal; GPa, Gigapascal; VHN, Vickers hardness number.

**Table 3:** One-way ANOVA of flexural strength, flexural modulus, Vickers microhardness, sorption, and solubility of study groups.

Property	Mean square	F test	*P*	*R* ^2^_adj_
Flexural strength	26.66	1.10	0.35	0.08
Flexural modulus	9.16	18.1	< 0.0001*	0.57
Vickers microhardness	1370.69	29.25	< 0.0001*	0.68
Sorption	4.04	17.67	< 0.0001*	0.57
Solubility	114.46	622.15	< 0.0001*	0.98

R^2^_adj_, adjusted R squared. *Statistically significant difference (*P* ≤ 0.05).

### Vickers Micro_Hardness results

**Fig. 6:**
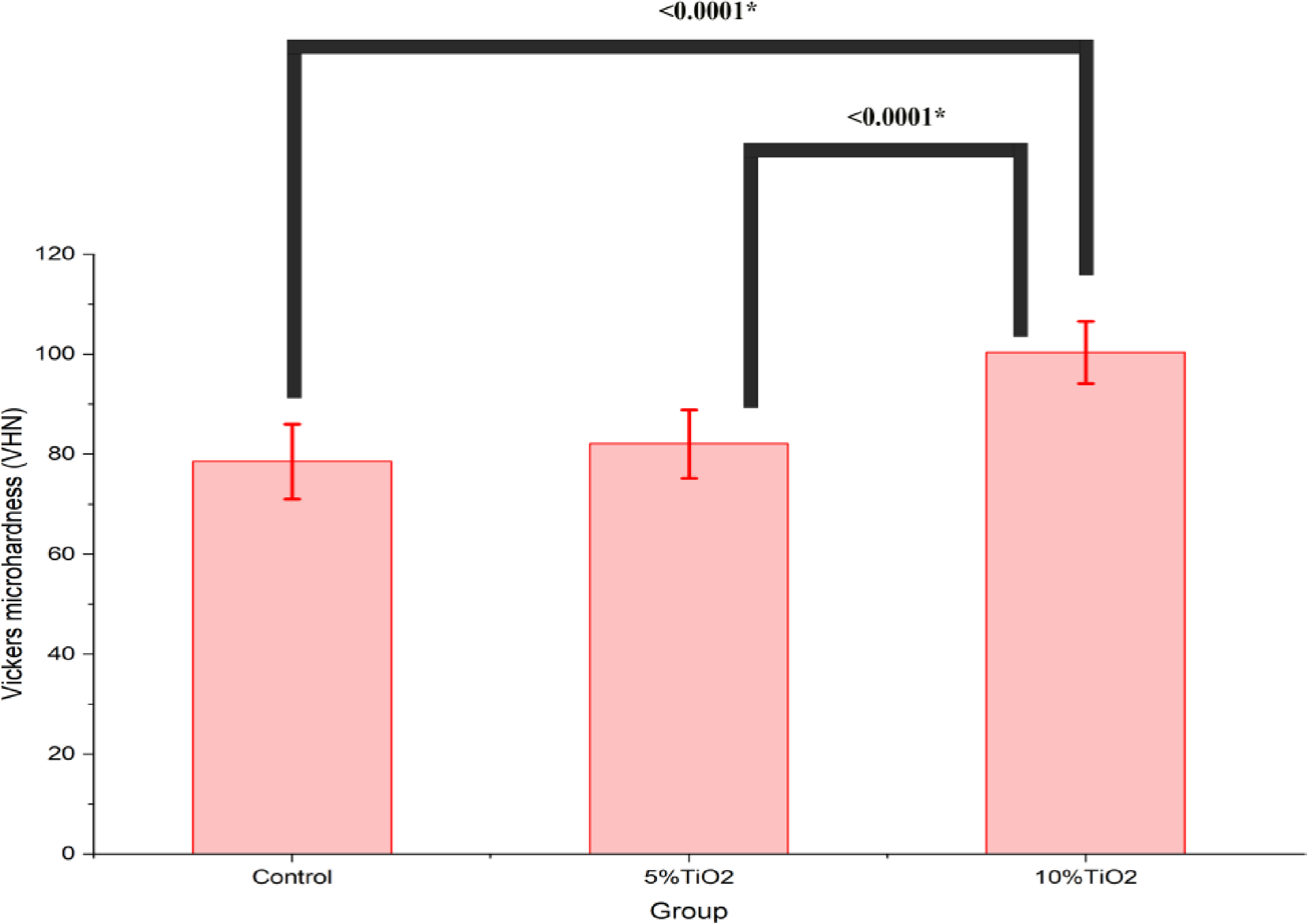
Comparisons of mean and SD of Vickers microhardness (VHN) between study groups.

Regarding microhardness, the 10%TiO_2_ group expressed superior performance at 100.4 ± 5.9 VHN (Table 2) with a significant difference compared to control group (*P* < 0.0001) and the 5% CA-TiO₂ NPs group (*P* < 0.0001) (Table 3**and** Fig. 6).

### Water sorption and solubility

**Fig. 7:**
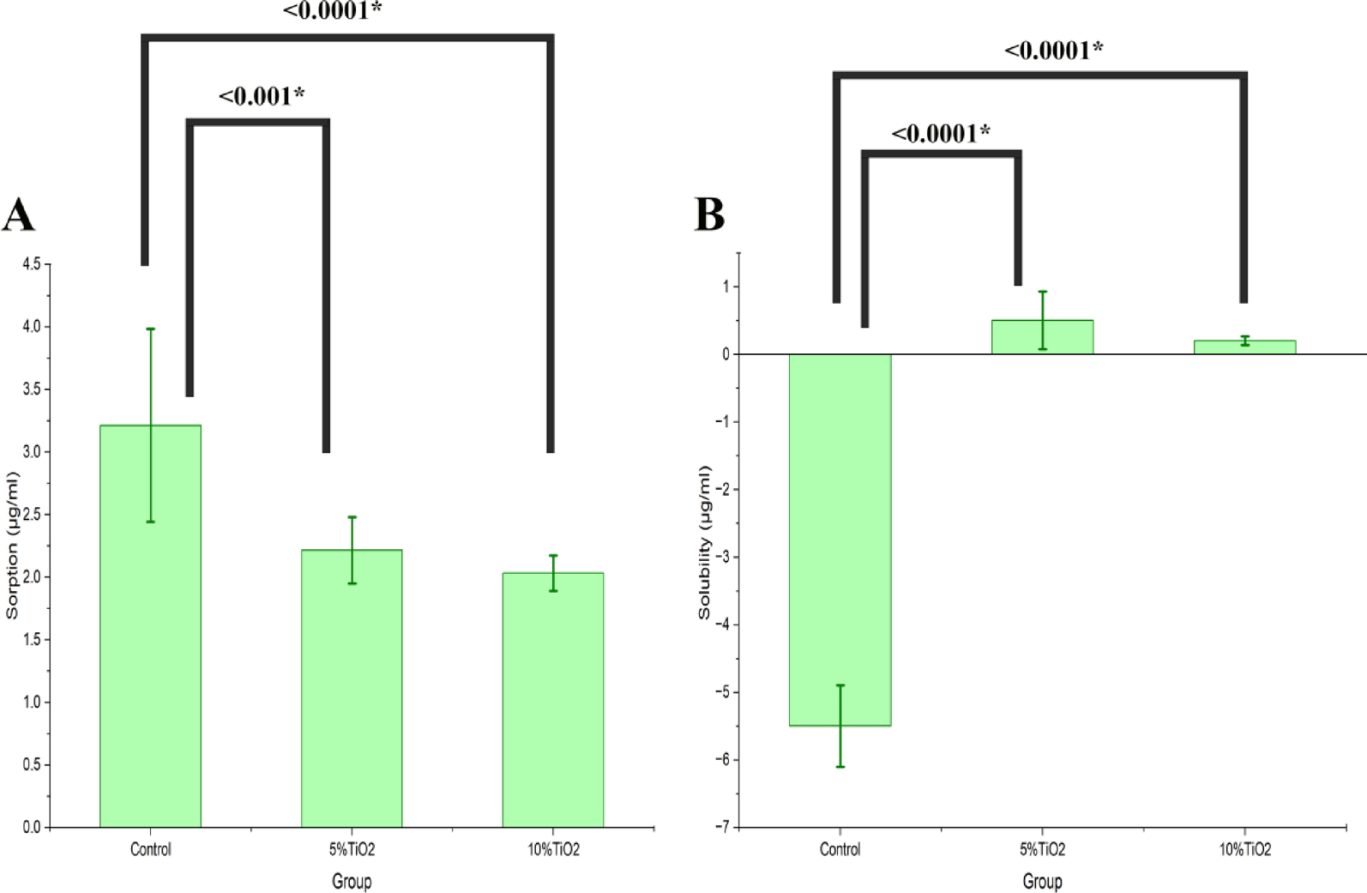
Comparisons of mean and SD of **(A)** sorption (µg/ml) and **(B)** solubility (µg/ml) between study groups.

As for the sorption behavior (Table 2), the control group had the highest sorption value after 7 days (3.2 ± 0.7 µg/ml), while the least value belonged to the 10%TiO2 group (2.0 ± 0.1 µg/ml) (Table 3**and** Fig. 7A). The difference between the control group and the 5% CA-TiO₂ NPs was significant at *P* < 0.001 and that between the control and the 10%TiO_2_ group was significant at *P* < 0.0001. Solubility values showed a similar pattern to sorption (Table 2), where the control group expressed a negative solubility of −5.5 ± 0.6 µg/ml which was significantly different compared to the other groups (*P* < 0.0001) (Table 3**and** Fig. 7B).

### In Silico results

#### SwissADME Results

**Fig. 8:**
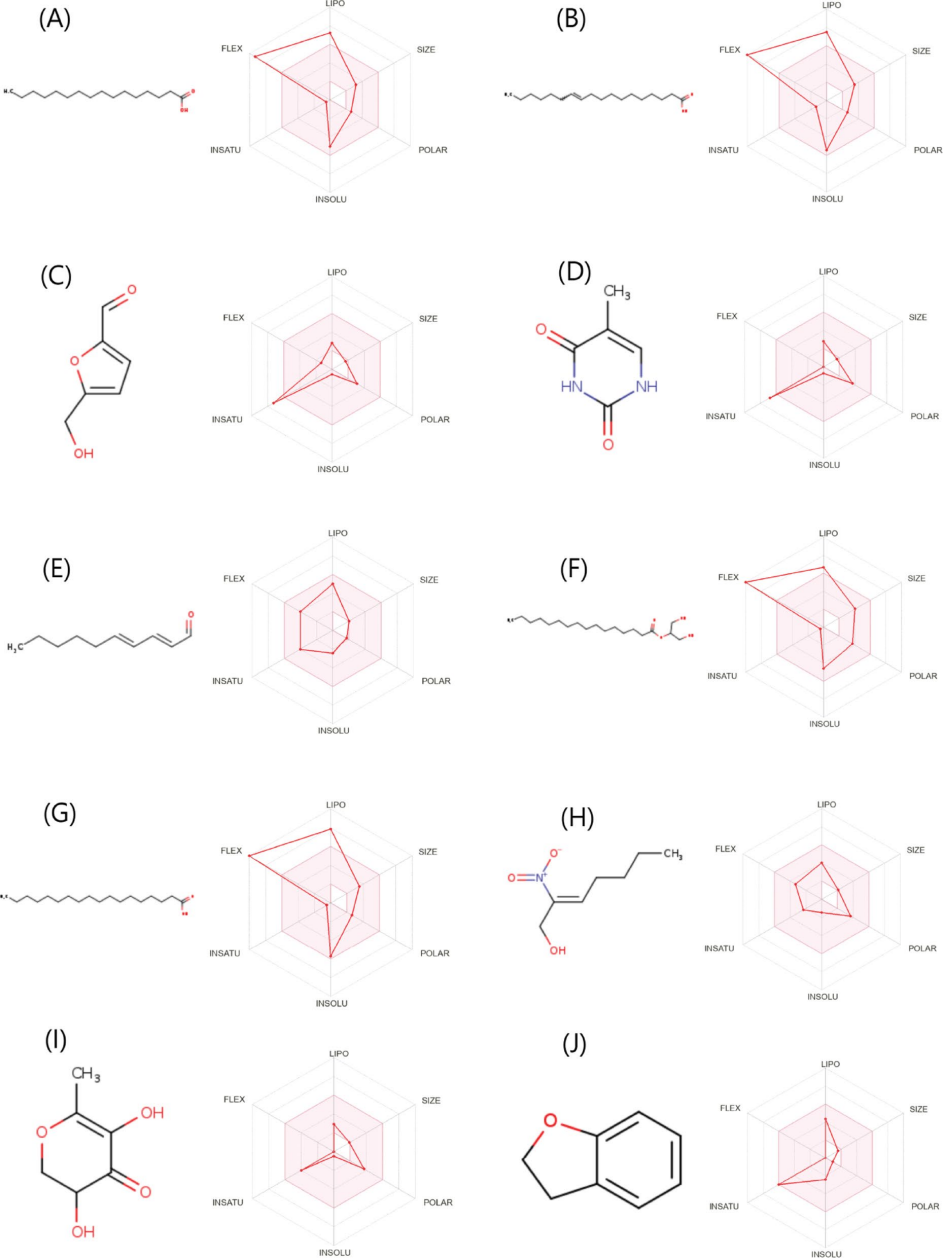
SwissADME “bioavailability radar” plots with 2D structures for the ten major GC-MS constituents identified in the *Citrus aurantium* seed extract examined in this study and used as screening-level physicochemical descriptors for the ten major GC-MS metabolites, reported to support hypothesis-generating hazard prioritization under an oral small-molecule model. The six axes report key descriptors: lipophilicity (LIPO), size or molecular weight (SIZE), polarity or TPSA (POLAR), insaturation or degree of unsaturation (INSATU), molecular flexibility or rotatable bonds (FLEX), and insolubility or aqueous solubility class (INSOLU). For each molecule, the red polygon shows the calculated properties and the pink zone indicates the preferred physicochemical space for oral bioavailability. These SwissADME outputs were generated from PubChem SMILES. Compound labels: **(A)** n-Hexadecanoic acid, **(B)** cis-Vaccenic acid, **(C)** 2-Furancarboxaldehyde 5-(hydroxymethyl)-, **(D)** Thymine, **(E)** 2,4-Decadienal (E, E), **(F)** Hexadecanoic acid 2-hydroxy-1-(hydroxymethyl)ethyl methyl ester, **(G)** Octadecanoic acid, **(H)** 2-Nitrohept-2-en-1-ol, **I** 4 H-Pyran-4-one 2,3-dihydro-3,5-dihydroxy-6-methyl-, **(J)** Benzofuran 2,3-dihydro-. (available under CC-BY 4.0 license).

SwissADME profiling of the ten GC–MS–identified constituents revealed two consistent chemotypes that contextualize the CA–TiO₂ NPs –GIC findings **(**Fig. 8A-J**)**: highly lipophilic fatty acids (consensus logP ≈ 4.7–5.9; poor-to-moderate solubility; 14–18 rotors) and small oxygenated heterocycles (consensus logP − 0.22 to 2.00; very high aqueous solubility; 0–2 rotors). All compounds showed High GI absorption (10/10), no P-gp substrate flags (10/10), and limited CYP liabilities—CYP1A2 inhibition in 4/10, CYP2C9 in 2/10, CYP2D6 in 1/10; CYP2C19 and CYP3A4 were uniformly No—supporting a low drug–drug interaction signal at likely leachable doses. BBB permeation was predicted for 5/10, but together with low bioavailability scores for several small molecules and high lipophilicity/low solubility for the fatty acids, these data argue for predominantly local action with limited systemic exposure. Medicinal chemistry filters were reassuring PAINS = 0 for all, with Brenk alerts confined to expected structural motifs (isolated alkene, aldehyde/michael acceptor, nitro).

#### Deep-PK Results

Deep-PK profiling of the ten GC–MS–identified phytochemicals provided exposure- and safety-relevant context for the CA-TiO₂ NPs –modified GIC. All compounds were predicted absorbed in the intestine (10/10), yet oral bioavailability > 50% was observed for only 6/10, and half-life > 3 h for 6/10, with clearance spanning − 0.78 to 11.58 log ml·min⁻¹·kg⁻¹. Together, these trends indicate that several constituents would be cleared rapidly if swallowed, while a subset may persist longer, supporting a primarily local rather than systemic contribution at clinical use levels. Although all molecules were flagged as BBB-penetrable in the model, the combination of modest bioavailability and, for many, higher clearance argues against meaningful central exposure at trace leachable doses. Importantly, CYP3A4 substrate status was negative for all compounds (10/10), and hERG blockade was not predicted (10/10 Safe), aligning with a low cardiac and metabolic-interaction liability. Safety endpoints showed AMES toxicity in 3/10, carcinogenicity in 3/10, and DILI-I in 2/10, identifying candidates for targeted follow-up while the majority remained Safe across these assays.

#### ProTox-3.0 Results

Most compounds fell in WHO acute toxicity Classes 4–5 (5/10 and 4/10, respectively), with LD₅₀ spanning 48–5000 mg/kg (median ≈ 1833 mg/kg), indicating moderate to low acute toxicity overall; a single Class 2 outlier was detected. Organ-toxicity calls were largely benign (hepatotoxicity, immunotoxicity, cytotoxicity: 0/10 Active). Limited signals were seen for neurotoxicity (2/10), nephrotoxicity (3/10) and cardiotoxicity (1/10), while carcinogenicity (4/10) and mutagenicity (2/10) flagged a minority of molecules for targeted follow-up. The BBB-barrier model was Active in 10/10, a conservative signal that we interpret cautiously given the expected low systemic exposure from the set material. Ecotoxicity was Active in 6/10, supporting responsible waste handling. Selected nuclear-receptor/stress readouts (PPAR-γ, nrf2/ARE, HSE, MMP) were each Active in 1/10. Overall, the model outputs suggest no widespread high-risk acute toxicity alerts across most molecules, while identifying a minority of endpoints (for example mutagenicity or ecotoxicity flags) that warrant targeted follow-up in experimental assays.

## Discussion

This study demonstrated that the addition of *Citrus aurantium*–mediated titanium dioxide nanoparticles (CA-TiO₂ NPs) substantially enhanced the mechanical and physical properties of glass ionomer cement (GIC). The integration of in silico analyses into this work provided molecular-level insight into the safety, pharmacokinetic, and physicochemical behavior of the phytoconstituents involved in nanoparticle synthesis, providing early-stage molecular safety context alongside the materials performance results.

Although phytochemical constituents were characterized in detail, their role in the present study was limited to mediating the synthesis of TiO₂ nanoparticles. These compounds were not introduced as free additives into the glass ionomer cement; therefore, the observed changes in material properties are attributed to the presence and distribution of TiO₂ nanoparticles rather than to direct phytochemical effects.

Flexural strength was in focus of the present study because this test permits the simulation of clinical loading conditions by providing an appropriate estimate of a material’s tensile strength, while improvements in flexural strength are closely linked to the microstructure integration^56^. Glass ionomer is thus subjected to flexural strength and elastic modulus evaluations which indicate the capacity of restorative material to resist high forces during the chewing process as well as prevent microleakage^57^. Furthermore, a three-point bending test is conducted in this study as per the ISO standard 9917-2:2017. It is regarded as a simulation of clinical situations involving the forces applied by opposing cusp. Moreover, it is the recommended test for evaluating the polymer-based materials^58^.

Importantly, no statistically significant differences in flexural strength were observed among the tested groups, indicating that TiO₂ nanoparticle incorporation did not enhance bulk fracture resistance of the glass ionomer cement. In general, the size of and interconnections among NPs during the mixing process play a crucial role in enhancing the GIC flexural strength^59^. Their small size likely allows NPs to fill larger voids in the material, thereby increasing its overall strength^60^. Generally, as the amount of TiO_2_ NPs increases, so does their number and surface area. However, when the amount of GIC particles is insufficient to bind with the TiO_2_ NPs, a larger number of free and unattached TiO_2_ nanoparticles will remain within the restorative material, reducing its flexural strength^61^.

The green synthesis method in this study yielded quasi-spherical nanoparticles averaging ~ 12 nm in size with crystalline anatase phase confirmed by XRD and TEM, promoting effective integration consistent with findings emphasizing nanoparticle morphology as critical to mechanical property enhancements. The improvement of flexural strength with addition of TiO_2_ NPs may be attributed to improved nanoparticle dispersion and matrix densification^61^. The values obtained for flexural strength in this study are also consistent with the global mean values for GIC flexural strength which are reported by *Morberg et al. to be around 18 to 30 MPa*^62^.

The findings of this study agree well with previous research reporting TiO_2_ NPs reinforcement in GICs. Notably, *Fathi et al.* (2022)^63^ found that 3–5 wt% TiO_2_ NPs incorporation increased surface microhardness and significantly decreased water sorption and solubility, although flexural strength improvements were not statistically significant at those lower concentrations. These results parallel the trend observed herein, where a higher concentration of 10 wt% TiO_2_ NPs achieved higher flexural strength gains albeit not statistically significant. Similarly, another study reported that TiO_2_ NPs at 5 wt% increased flexural and diametral tensile strengths, attributing the improvements to covalent bonding between surface hydroxyl groups of TiO_2_ and the GIC matrix as well as nanoparticle filling of microscopic voids, reinforcing the material structure^59^.

The elastic modulus represents the relationship between the stress applied to a material and the resulting deformation it undergoes^64^. Ideally, restorative materials should have an elastic modulus comparable to that of natural dental tissues and low enough to absorb functional stresses without causing cusp fractures^65^. For posterior restorations, the material should have an elastic modulus equal to or higher than that of dentin, which ranges from approximately 5–13 GPa^66^.

In the present study, the incorporation of TiO₂NPs into GIC resulted in higher elastic modulus values compared with the control group. Since different clinical situations demand restorative materials with varying elastic moduli, the statistically significant differences observed, particularly between the filled groups and the control, indicate that GIC modified with 5% and 10% CA-TiO₂ NPs may be suitable for high-stress areas subjected to strong occlusal forces.

Although the addition of the NPs had a non-significant change on flexural strength, it had a significant difference over the flexural modulus. This can be attributed to the dependence of flexural strength on the adhesion between matrix and filler, contrary to the elastic modulus which does not rely on filler adhesion to matrix. Therefore, the addition of higher amounts of filler would help fill the voids between the resin chains and reduce their movement, thus enhancing elastic modulus, while these unbound particles can lead to a decline in flexural strength^67^.

Surface hardness is a property that indicates resistance of a material to penetration or indentation, which also reflects the material’s ability to resist wear and abrasion during prolonged intraoral use^68^. The addition of TiO_2_ NPs to GIC enhanced its microhardness, especially when higher loading percentages were employed (10%). The small filler size helped increase the surface area and interfacial energy binding the particles to the GIC matrix leading to a more coherent interface with less gaps, thus resisting surface penetration^69^. Another contributing factor is perhaps the presence of more TiO_2_ NPs than glass on the surface leading to the formation of a stable complex upon interaction of the particles with polyacrylic acid. Such a complex improved colloidal stability and prevented particle agglomeration, therefore contributing to the overall surface stability^57,70^.

Results of this study are in line with Fathi et al. who added 3 and 5% of CA-TiO₂ NPs to GIC which improved microhardness significantly compared to unfilled GIC^63^. Also, Garcia-Contreras et al. found a significant increase in microhardness when CA-TiO₂ NPs were added in the ratios of 3 and 5% to restorative GIC resin^57^.

Water sorption characteristics and solubility are critical parameters for evaluating the clinical performance of dental cements. GICs are highly sensitive to water presence in the first 24 h. The large amount of water sorption changes the material volume and deteriorates the matrix structure^71^. It affects properties such as strength, hardness, flexion and mechanical stability. The absorbed water reacts with the material particles leading to their separation and contributing to their release, which increases material solubility^71^. This is evident in the negative solubility values of the control group which are likely to reflect water uptake exceeding mass loss due to the post-curing water uptake phenomenon which is common in resinous porous materials^72^. Additionally, this might be attributed to the water retention in the matrix where it is bound to the resin chains and cannot be removed despite thorough dehydration of the specimens^73^.

The GIC modified with 10% TiO₂ nanoparticles exhibited the lowest solubility and sorption values. This improvement in physical properties can be attributed to the low solubility of the nanoparticles in aqueous media, allowing them to occupy the voids between the larger glass particles within the GIC. These nanoparticles also provide additional binding sites for polyacrylic acid, contributing to a more reinforced and stable GIC matrix^74^.

Water sorption and solubility reductions observed here align with several reports highlighting improved dimensional stability of nanoparticle-modified GICs due to the hydrophobic nature of TiO_2_ and its pore-sealing effect. Stability against aqueous degradation is essential for enhancement of clinical performance, reinforcing the practical significance of these modifications^65^.

Integration of in silico results deepened understanding of the material’s biocompatibility and environmental safety. SwissADME and Deep-PK screening revealed that the identified metabolites exhibited high gastrointestinal absorption, no P-gp-related liabilities, and low molecular flexibility, suggesting restricted systemic diffusion in case of accidental leachability. ProTox-3.0. predicted WHO Class IV–V toxicity levels (LD₅₀ > 1000 mg/kg) indicated low acute and organ toxicity, reinforcing the hypothesis that the natural metabolites used in nanoparticle synthesis pose minimal biological risk. Together, these computational outputs complemented the experimental observations, supporting that the observed mechanical strengthening is not accompanied by major predicted toxicity alerts under a hypothetical leachate exposure screen.

Specific metabolites such as n-hexadecanoic acid and thymine demonstrated low reactivity and negligible PAINS alerts, confirming that they can act as benign, stabilizing surface agents rather than chemically active leachables. Such molecular properties explain the observed stability of the CA-TiO₂ NPs –modified GIC and suggest that the biogenic capping molecules not only guide nanoparticle formation but also enhance interfacial compatibility.

This cross-linking of experimental and computational evidence establishes a novel paradigm for designing green restorative materials. Unlike earlier studies that emphasized only mechanical reinforcement, this hybrid approach provides preliminary, screening-level safety context for green nanoparticles and helps prioritize endpoints for downstream experimental validation. The dual verification, experimental durability and computational toxicity profiling, enhances translational confidence in their clinical application.

### Study Limitations

Nevertheless, this investigation had limitations. Only two concentrations of TiO_2_ NPs were evaluated which can have a significant yet variable effect on physical properties. However, it is observed in some pilot tests that by increasing the NPs percentage, the samples become fragile. *Ma et al.* ^75^, demonstrated that using concentrations below 1% did not lead to a reduction in physical properties and did not exhibit any significant influence on the GICs cytotoxicity, which is an important requirement that any dental tooth filling material must meet. Besides, the NPs concentration in the final composite must be carefully selected as higher concentrations can alter the bond quality with dentin^76^. Therefore, it was decided to use 5% and 10% in both experimental groups. The in silico evaluation modeled isolated phytochemicals without considering the complex networking within the cement environment. Because SwissADME, Deep-PK, and ProTox are primarily trained and validated for freely dissolved small molecules under oral exposure assumptions, the present analysis was used strictly as screening-level toxicoinformatics on the identified metabolites^77–79^. The models do not capture immobilization within a glass ionomer matrix, mixture effects, or release kinetics. Therefore, outputs are interpreted as intrinsic hazard descriptors under a hypothetical exposure scenario and are used to prioritize subsequent leachate and cytotoxicity testing. Additionally, no antibacterial or biofilm assays were performed to test the effectiveness of these nanoparticles as anticariogenic agents.

Further work is required to experiment with different concentrations and assess the effects of aging on the properties of titanium dioxide reinforced GIC. Also, Future studies should integrate cytotoxicity assays and dynamic ion-release analysis to correlate molecular predictions with biological outcomes under oral-like conditions^80^. Broader computational modeling that includes protein-ligand and salivary interaction simulations could further refine safety profiles.

## Conclusions

The integration of green-synthesized titanium dioxide nanoparticles, obtained through *Citrus aurantium* seed extract, can enhance elastic modulus, surface microhardness, and resistance to water sorption and solubility. The improvement of such properties would allow the use of GIC in load-bearing areas and provide higher abrasion resistance as well as restoration stability enhancement. Beyond experimental evaluation, the integration of in silico pharmacokinetic and toxicological screening provided a complementary, early-stage safety context for the phytochemical constituents associated with nanoparticle synthesis. These findings support a proof-of-concept framework for integrating green nanotechnology with preliminary computational safety screening, warranting further biological and long-term validation and provide useful preliminary insights into physicochemical behavior and potential toxicological alerts.

## Supplementary Information

Below is the link to the electronic supplementary material.

Supplementary Material 1.

Supplementary Material 2.

## Data Availability

All data generated or analysed during this study are included in this published article and its sup-plementary information files.

## References

[1] Sidhu, S. K. & Nicholson, J. W. A review of Glass-Ionomer cements for clinical dentistry. *J. Funct. Biomater.* **7**10.3390/jfb7030016 (2016). 10.3390/jfb7030016 PMC5040989 27367737

[2] Hamdy, T. M. Evaluation of compressive strength, surface microhardness, solubility and antimicrobial effect of glass ionomer dental cement reinforced with silver doped carbon nanotube fillers. *BMC Oral Health*. **23**, 777. 10.1186/s12903-023-03542-6 (2023). 37872523 10.1186/s12903-023-03542-6 PMC10591371

[3] Bali, P., Prabhakar, A. R. & Basappa, N. An Invitro Comparative Evaluation of Compressive Strength and Antibacterial Activity of Conventional GIC and Hydroxyapatite Reinforced GIC in Different Storage Media. *J Clin Diagn Res* 9, Zc51-55, (2015). 10.7860/jcdr/2015/13012.6205 10.7860/JCDR/2015/13012.6205 PMC4573037 26393206

[4] Ezzat, D. et al. Phytomedicine and green nanotechnology: enhancing glass ionomer cements for sustainable dental restorations: a comprehensive review. *Beni-Suef Univ. J. Basic. Appl. Sci.* **14**, 48. 10.1186/s43088-025-00633-x (2025).

[5] Azab, A. et al. Systematic review and meta analysis of mechanical properties of 3D printed denture bases compared to milled and conventional materials. *Sci. Rep.* **15**, 29207. 10.1038/s41598-025-14288-2 (2025). 40783443 10.1038/s41598-025-14288-2 PMC12335478

[6] Azab, A., El-Sheikh, A. & Abd-Allah, S. Stress analysis of short implants with different diameters in maxillary bilateral distal extension bases. *Tanta Dent. J.* **17**, 73. 10.4103/tdj.tdj_55_19 (2020).

[7] Azab, A., Alam-Eldein, A. & Aboutaleb, F. Qualitative and radiographic assessment of PEEK bar versus titanium bar fabricated by CAD-CAM in mandibular hybrid prosthesis: a randomized controlled clinical trial. *Ain Shams Dent. J.* **35**, 334–343. 10.21608/asdj.2024.291266.1289 (2024).

[8] Alsuraifi, A. et al. State of art: a narrative review on navigating pathogenesis and therapies for genetic oral disorders. *Frontiers Oral Maxillofacial Medicine* **7** (2024).

[9] Alsuraifi, A. et al. Revolutionizing tooth regeneration: innovations from stem cells to tissue engineering. *Regenerative Eng. Translational Med.* **11**, 625–650. 10.1007/s40883-024-00382-w (2025).

[10] Abdelhady, W. et al. A systematic review on influence of printing layer thickness on the marginal and internal fit of 3D-printed fixed dental prostheses. *Odontology*10.1007/s10266-025-01241-y (2025). 41264112 10.1007/s10266-025-01241-y PMC13320041

[11] Takahashi, Y. et al. Antibacterial effects and physical properties of glass-ionomer cements containing chlorhexidine for the ART approach. *Dent. Mater.* **22**, 647–652. 10.1016/j.dental.2005.08.003 (2006). 16226806 10.1016/j.dental.2005.08.003

[12] Toledano, M. et al. Sorption and solubility of resin-based restorative dental materials. *J. Dent.* **31**, 43–50. 10.1016/s0300-5712(02)00083-0 (2003). 12615019 10.1016/s0300-5712(02)00083-0

[13] Hajmiragha, H., Nokar, S., Alikhasi, M., Nikzad, S. & Dorriz, H. Solubility of three Luting cements in dynamic artificial saliva. *Journal Dentistry Tehran Univ. Med. Sciences* **5** (2008).

[14] Mitra, S. B. & Kedrowski, B. L. Long-term mechanical properties of glass ionomers. *Dent. Mater.* **10**, 78–82. 10.1016/0109-5641(94)90044-2 (1994). 7758852 10.1016/0109-5641(94)90044-2

[15] Abozaid, D. et al. Bioactive restorative materials in dentistry: a comprehensive review of mechanisms, clinical applications, and future directions. *Odontology*10.1007/s10266-025-01162-w (2025). 40819015 10.1007/s10266-025-01162-w PMC13053330

[16] Alsuraifi, A. et al. Explore the most recent developments and upcoming outlooks in the field of dental nanomaterials. *Beni-Suef Univ. J. Basic. Appl. Sci.* **13**10.1186/s43088-024-00540-7 (2024).

[17] Ezzat, D., Sheta, M. S., Kenawy, E. R., Eid, M. A. & Elkafrawy, H. Synthesis, characterization and evaluation of experimental dental composite resin modified by grapefruit seed extract-mediated TiO₂ nanoparticles: green approach. *Odontology* **113**, 1148–1164. 10.1007/s10266-025-01058-9 (2025). 39961895 10.1007/s10266-025-01058-9 PMC12178990

[18] Lv, X. et al. Citrus fruits as a treasure trove of active natural metabolites that potentially provide benefits for human health. *Chem. Cent. J.* **9**, 1–14 (2015). 26705419 10.1186/s13065-015-0145-9 PMC4690266

[19] Liu, Y., Heying, E. & Tanumihardjo, S. A. History, global distribution, and nutritional importance of citrus fruits. *Compr. Rev. Food Sci. Food Saf.* **11**, 530–545 (2012).

[20] Mannucci, C. et al. Clinical Pharmacology of Citrus aurantium and Citrus sinensis for the Treatment of Anxiety. *Evidence-based complementary and alternative medicine: eCAM* (2018). (2018). 10.1155/2018/3624094 PMC6304613 30622597

[21] Westanmo, A. Citrus aurantium. *Herbal Products: Toxicol. Clin. Pharmacology*, 233–244 (2007).

[22] Maksoud, S. et al. Citrus aurantium L. Active Constituents, biological effects and extraction methods. *Updated Rev. Molecules*. **26**, 5832 (2021). 10.3390/molecules26195832 PMC8510401 34641373

[23] Bachhar, V. et al. Green synthesis of AgFe bimetallic nanoparticles from calyptocarpus Vialis plant extract for enhanced catalytic reduction of 4-NP, antioxidant and antibacterial activities. *J. Environ. Chem. Eng.* **13**, 116829. 10.1016/j.jece.2025.116829 (2025).

[24] Bachhar, V., Joshi, V. & Antibacterial Antioxidant, and antidiabetic activities of TiO2 nanoparticles synthesized through ultrasonication assisted cold maceration from stem extract of euphorbia Hirta. *Lett. Appl. Nanobiosci.* **14**10.33263/LIANBS141.001 (2024).

[25] Joshi, V. et al. Sustainable valorisation of onion Peel waste through silver nanoparticle synthesis: Antibacterial, antidiabetic and computational insights. *J. Mol. Struct.* **1352**, 144570. 10.1016/j.molstruc.2025.144570 (2026).

[26] Bachhar, V. et al. Green synthesis of TiO2/Ag/Cu2O nanoparticles: antioxidant potential and in-vitro/in-vivo antidiabetic activity in STZ-induced diabetic albino mice. *Inorg. Chem. Commun.* **183**, 115900. 10.1016/j.inoche.2025.115900 (2026).

[27] Joshi, V. et al. Bioactive compounds of Piper Chaba and their Pharmacological relevance: a phytochemistry-focused review. *Phytochem. Rev.*10.1007/s11101-025-10193-8 (2025).

[28] Alsuraifi, A. et al. Quantum dots: catalysts for a new era of precision medicine and biomedical innovation. *J. Fluoresc*. 10.1007/s10895-025-04452-2 (2025). 41051600 10.1007/s10895-025-04452-2

[29] Joshi, V. et al. GC–MS fingerprinting, nutritional composition, in vitro Pharmacological activities and molecular Docking studies of Piper Chaba from Uttarakhand region. *3 Biotech.* **14** (158). 10.1007/s13205-024-03996-7 (2024). 10.1007/s13205-024-03996-7 PMC11101386 38766322

[30] Bachhar, V., Joshi, V., Gangal, A., Duseja, M. & Shukla, R. K. Identification of bioactive Phytoconstituents, nutritional composition and antioxidant activity of calyptocarpus Vialis. *Appl. Biochem. Biotechnol.* **196**, 1921–1947. 10.1007/s12010-023-04640-5 (2024). 37450214 10.1007/s12010-023-04640-5

[31] Ashour, A. A. et al. Antimicrobial efficacy of glass ionomer cement in incorporation with biogenic Zingiber officinale capped Silver-Nanobiotic, chlorhexidine diacetate and lyophilized Miswak. *Molecules* **27**10.3390/molecules27020528 (2022). 10.3390/molecules27020528 PMC8781574 35056835

[32] Ilancheran, P., Paulraj, J., Maiti, S. & Shanmugam, R. Green Synthesis, Characterization, and evaluation of the antimicrobial properties and compressive strength of hydroxyapatite Nanoparticle-Incorporated glass ionomer cement. *Cureus* **16**, e58562. 10.7759/cureus.58562 (2024). 38770461 10.7759/cureus.58562 PMC11102871

[33] Siddiqui, A., Gul, A., Khan, H., Anjum, F. & Hussain, T. Bio-inspired synthesis of silver nanoparticles usingsalsola imbricataand its application as antibacterial additive in glass ionomer cement. *Nanotechnology* **35**10.1088/1361-6528/ad50e4 (2024). 10.1088/1361-6528/ad50e4 38806018

[34] Daina, A., Michielin, O. & Zoete, V. SwissADME: a free web tool to evaluate pharmacokinetics, drug-likeness and medicinal chemistry friendliness of small molecules. *Sci. Rep.* **7**, 42717. 10.1038/srep42717 (2017). 28256516 10.1038/srep42717 PMC5335600

[35] Myung, Y., de Sá, A. G. C. & Ascher, D. B. Deep-PK: deep learning for small molecule Pharmacokinetic and toxicity prediction. *Nucleic Acids Res.* **52**, W469–w475. 10.1093/nar/gkae254 (2024). 38634808 10.1093/nar/gkae254 PMC11223837

[36] Drwal, M. N., Banerjee, P., Dunkel, M., Wettig, M. R. & Preissner, R. ProTox: a web server for the in Silico prediction of rodent oral toxicity. *Nucleic Acids Res.* **42**, W53–58. 10.1093/nar/gku401 (2014). 24838562 10.1093/nar/gku401 PMC4086068

[37] Abozaid, D., Ayad, A. & Azab, A. Enhancing glass ionomer cement with citrus aurantium L. extract: a combined in vitro and in Silico investigation of antimicrobial and mechanical properties. *Beni-Suef Univ. J. Basic. Appl. Sci.* **14**, 117. 10.1186/s43088-025-00701-2 (2025).

[38] Tiwari, A. K. et al. Green synthesis of TiO2 nanoparticles using Kinnow Peel extracts and their antioxidant properties. *Sci. Rep.* **15**, 38307. 10.1038/s41598-025-22078-z (2025). 41184334 10.1038/s41598-025-22078-z PMC12583586

[39] Mobeen Amanulla, A. & R, S. Green synthesis of TiO2 nanoparticles using orange peel extract for antibacterial, cytotoxicity and humidity sensor applications. *Materials Today: Proceedings* 8, 323–331, (2019). 10.1016/j.matpr.2019.02.118

[40] Lawal, A. et al. Phytochemical analysis and thin layer chromatography profiling of crude extracts from Senna occidentalis (leaves). *J. Biotechnol. Biomedical Sci.* **2**, 12–21 (2019).

[41] Firdouse, S., Alam, P. & J. I. J. o. P. Phytochemical investigation of extract of amorphophallus campanulatus tubers.*Int. J. Phytomedicine* **3**, 32 (2011).

[42] Purewal, S. S. & J. J. N. P. P. R. Phytochemical analysis of ethanolic extracts of different pearl millet (Pennisetum glaucum) varieties. *Agric. Food Sci.* **4**, 19–23 (2014).

[43] Raphael, E. J. G. A. R. J. o. E. S. & Toxicology. Phytochemical constituents of some leaves extract of Aloe vera and Azadirachta indica plant species.*Glob. Adv. Res. J. Environ. Sci. Toxicol.* **1**, 014–017 (2012).

[44] Dubale, S., Kebebe, D., Zeynudin, A., Abdissa, N. & Suleman, S. Phytochemical screening and antimicrobial activity evaluation of selected medicinal plants in Ethiopia. *J. Exp. Pharmacol.* **15**, 51–62. 10.2147/jep.S379805 (2023). 36789235 10.2147/JEP.S379805 PMC9922502

[45] Asadollahi-Baboli, M. & Aghakhani, A. Rapid analysis of O riganum Majorana L. fragrance using a nanofiber sheet, gas chromatography with mass spectrometry, and chemometrics. *J. Sep. Sci.* **37**, 990–996 (2014). 24520037 10.1002/jssc.201301355

[46] Eneke, I. C., Essien, E. B. & Wegu, M. O. Gas chromatography-Mass spectrometry (GC-MS) analysis of bioactive components present in grape citrus Peel in Nigeria. *GSC Adv. Res. Reviews*. **8**, 166–174 (2021).

[47] Zeebaree, A. et al. Sustainable engineering of plant-synthesized TiO2 nanocatalysts: diagnosis, properties and their photocatalytic performance in removing of methylene blue dye from effluent. A review. *Curr. Res. Green. Sustainable Chem.* **5**, 100312. 10.1016/j.crgsc.2022.100312 (2022).

[48] Mousa, H. et al. Development of environmentally friendly catalyst Ag-ZnO@cellulose acetate derived from discarded cigarette butts for reduction of organic dyes and its antibacterial applications. *Int. J. Biol. Macromol.* **258**, 128890. 10.1016/j.ijbiomac.2023.128890 (2024). 38134996 10.1016/j.ijbiomac.2023.128890

[49] Aldosari, B. N. et al. Synthesis and characterization of magnetic Ag-Fe(3)O(4)@polymer hybrid nanocomposite systems with promising antibacterial application. *Drug Dev. Ind. Pharm.* **49**, 723–733. 10.1080/03639045.2023.2277812 (2023). 37906615 10.1080/03639045.2023.2277812

[50] Bhullar, S., Goyal, N. & Gupta, S. A recipe for optimizing TiO2 nanoparticles for drug delivery applications. *OpenNano* **8**, 100096. 10.1016/j.onano.2022.100096 (2022).

[51] Rami, J. M., Patel, C. D., Patel, C. M. & Patel, M. V. Thermogravimetric analysis (TGA) of some synthesized metal oxide nanoparticles. *Mater. Today: Proc.* **43**, 655–659. 10.1016/j.matpr.2020.12.554 (2021).

[52] Abozaid, D. et al. Antimicrobial mechanical and molecular Docking analysis of dental composite resin incorporating green synthesized titanium dioxide nanoparticles from vitis vinifera extract. *Sci. Rep.* **15**, 35042. 10.1038/s41598-025-20989-5 (2025). 41062669 10.1038/s41598-025-20989-5 PMC12508154

[53] de Morais Sampaio, G. A. et al. Antimicrobial properties, mechanics, and fluoride release of ionomeric cements modified by red propolis. *Angle Orthod.* **91**, 522–527. 10.2319/083120-759.1 (2021). 33630071 10.2319/083120-759.1 PMC8259754

[54] Standard test method for microhardness of materials. American society for testing and materials annual book of standards. *Am. Soc. Test. Mater. Annual Book. Stand.* **3**, 384 (1999).

[55] Gonulol, N., Ozer, S. & Sen Tunc, E. Water Sorption, Solubility, and color stability of Giomer restoratives. *J. Esthet Restor. Dent.* **27**, 300–306. 10.1111/jerd.12119 (2015). 25145876 10.1111/jerd.12119

[56] Baig, M. S., Dowling, A. H., Cao, X. & Fleming, G. J. A discriminatory mechanical testing performance indicator protocol for hand-mixed glass-ionomer restoratives. *Dent. Mater.* **31**, 273–283. 10.1016/j.dental.2014.12.012 (2015). 25593050 10.1016/j.dental.2014.12.012

[57] Garcia-Contreras, R. et al. Mechanical, antibacterial and bond strength properties of nano-titanium-enriched glass ionomer cement. *J. Appl. Oral Sci.* **23**, 321–328. 10.1590/1678-775720140496 (2015). 26221928 10.1590/1678-775720140496 PMC4510668

[58] Organization., I. S. & Dentistry Polymer-based restorative materials. (ISO 4049:2009). (2009).

[59] Ramić, B. et al. Physical and mechanical properties assessment of glass ionomer cements modified with TiO2 and Mg-doped hydroxyapatite nanoparticles. *J. Appl. Biomater. Funct. Mater.* **22**, 22808000241282184. 10.1177/22808000241282184 (2024). 39413057 10.1177/22808000241282184

[60] Moheet, I. A. et al. Evaluation of mechanical properties and bond strength of nano-hydroxyapatite-silica added glass ionomer cement. *Ceram. Int.* **44**, 9899–9906 (2018).

[61] Amin, F. et al. Effect of nanostructures on the properties of glass ionomer dental Restoratives/Cements: A comprehensive narrative review. *Mater. (Basel)*. 14. 10.3390/ma14216260 (2021). 10.3390/ma14216260 PMC8584882 34771787

[62] Moberg, M., Brewster, J., Nicholson, J. & Roberts, H. Physical property investigation of contemporary glass ionomer and resin-modified glass ionomer restorative materials. *Clin. Oral Investig*. **23**, 1295–1308. 10.1007/s00784-018-2554-3 (2019). 29998443 10.1007/s00784-018-2554-3

[63] Fathi, U., Agha, M. & Ahmad, Z. The effect of the incorporation of titanium dioxide nanoparticles on the mechanical and physical properties of glass ionomer cement. *J. Res. Med. Dent. Sci.* **10**, 88-91 (2022).

[64] Baskar, H., Hari, A. & Anirudhan, S. Comparative evaluation of flexural Strength, modulus of Elasticity, and microleakage of three different glass ionomer restorative materials in class V Preparations – An in vitro study. *Indian J. Dent. Sci.* **15**, 67. 10.4103/ijds.ijds_54_22 (2023).

[65] Pérez-Castro, B. et al. Comparison of the physical properties of glass ionomer modified with silver phosphate/hydroxyapatite or titanium dioxide nanoparticles: in vitro study. *J. Clin. Pediatr. Dent.* **48**, 160–167. 10.22514/jocpd.2024.089 (2024). 39087226 10.22514/jocpd.2024.089

[66] Chun, K., Choi, H. & Lee, J. Comparison of mechanical property and role between enamel and dentin in the human teeth. *J. Dent. Biomech.* **5**, 1758736014520809. 10.1177/1758736014520809 (2014). 24550998 10.1177/1758736014520809 PMC3924884

[67] Zabihzadeh, S., Omidvar, A., Marandi, M., Mirmehdi, M. & Dastoorian, F. Physical and mechanical properties of rapeseed Waste-filled LLDPE composites. *J. Thermoplast. Compos. Mater.* **24**, 447–458. 10.1177/0892705710388591 (2011).

[68] Abozaid, D. et al. Effects of propolis-modified glass ionomer cement on antimicrobial activity and physico-mechanical properties: a systematic review. *Odontology*10.1007/s10266-025-01209-y (2025). 41026331 10.1007/s10266-025-01209-y PMC13053388

[69] Hepdeniz, Ö. K. & Gürdal, O. The effect of titanium dioxide nanoparticles on microhardness and SEM-EDS analysis of glass ionomer cement and Amalgomer. *Selcuk Dent. J.* **8**, 623–628 (2021).

[70] Kanehira, K. et al. Properties of TiO2–polyacrylic acid dispersions with potential for molecular recognition. *Colloids Surf. B Biointerfaces*. **64**, 10–15 (2008). 18296033 10.1016/j.colsurfb.2007.12.018

[71] Roberts, H., Berzins, D. & Nicholson, J. Long-Term water balance evaluation in glass ionomer restorative materials. *Mater. (Basel)*. **15**10.3390/ma15030807 (2022). 10.3390/ma15030807 PMC8836498 35160751

[72] Chung, D. D. L., Ozturk, M. & Xi, X. Drying-Participating water and drying-Induced air voids in the Post-curing drying of Cement-Based material. *Transp. Porous Media*. **153**, 2. 10.1007/s11242-025-02252-7 (2025).

[73] Ömeroğlu, M. K. & Hekimoğlu, H. C. Evaluation of colour stability, water sorption and solubility of no-cap flowable bulk fill resin composites. *BMC Oral Health*. **25**, 604. 10.1186/s12903-025-05902-w (2025). 40253330 10.1186/s12903-025-05902-w PMC12009527

[74] Genaro, L. E., Anovazzi, G., Hebling, J. & Zuanon, A. C. C. Glass ionomer cement modified by resin with incorporation of nanohydroxyapatite: in vitro evaluation of Physical-Biological properties. *Nanomaterials* **10**, 1412 (2020). 32707741 10.3390/nano10071412 PMC7408555

[75] Wang, J. et al. Inflammatory tumor microenvironment responsive neutrophil exosomes-based drug delivery system for targeted glioma therapy. *Biomaterials* **273**, 120784. 10.1016/j.biomaterials.2021.120784 (2021). 33848731 10.1016/j.biomaterials.2021.120784

[76] Abed, F. M. et al. Effect of different concentrations of silver nanoparticles on the quality of the chemical bond of glass ionomer cement dentine in primary teeth. *Front. Bioeng. Biotechnol.* **10**, 816652. 10.3389/fbioe.2022.816652 (2022). 35330624 10.3389/fbioe.2022.816652 PMC8940235

[77] Bachhar, V. et al. In-Vitro Antimicrobial, antidiabetic and anticancer activities of calyptocarpus Vialis extract and its integration with computational studies. *ChemistrySelect* **9**10.1002/slct.202401414 (2024).

[78] Joshi, V. et al. Identification of potential anticancer phytochemicals of Piper Chaba by comprehensive molecular Docking, molecular dynamics simulation and In-Vitro studies. *Chem. Biodivers.* **22**, e202403269. 10.1002/cbdv.202403269 (2025). 40101115 10.1002/cbdv.202403269

[79] Joshi, M. et al. Metabolite profiling and evaluation of antioxidant, antidiabetic, and antibacterial potential of thymus linearis Benth. Supported by molecular Docking and PASS prediction. *Sci. Rep.*10.1038/s41598-025-32317-y (2025). 41392186 10.1038/s41598-025-32317-y PMC12820053

[80] Joshi, V. et al. Bioactive compounds of Piper Chaba and their Pharmacological relevance: a phytochemistry-focused review. *Phytochem. Rev.* 1–66. 10.1007/s11101-025-10193-8 (2025).

